# Role of antiviral CD8+ T cell immunity to SARS-CoV-2 infection and vaccination

**DOI:** 10.1128/jvi.01350-24

**Published:** 2025-03-03

**Authors:** Vivien Karl, Maike Hofmann, Robert Thimme

**Affiliations:** 1 Department of Medicine II (Gastroenterology, Hepatology, Endocrinology and Infectious Diseases), Freiburg University Medical Center, Faculty of Medicine, University of Freiburg 9174 , Freiburg, Germany; 2 Faculty of Biology, University of Freiburg 9174 , Freiburg, Germany; New York University Department of Microbiology, New York, New York, USA

**Keywords:** CD8+ T cells, SARS-CoV-2, COVID-19, virus-specific immunity

## Abstract

The COVID-19 pandemic has greatly enhanced our understanding of CD8+ T cell immunity and their role in natural infection and vaccine-induced protection. Rapid and early SARS-CoV-2-specific CD8+ T cell responses have been associated with efficient viral clearance and mild disease. Virus-specific CD8+ T cell responses can compensate for waning, morbidity-related, and iatrogenic reduction of humoral immunity. After infection or vaccination, SARS-CoV-2-specific memory CD8+ T cells are formed, which mount an efficient recall response in the event of breakthrough infection and help to protect from severe disease. Due to their breadth and ability to target mainly highly conserved epitopes, SARS-CoV-2-specific CD8+ T cells are also able to cross-recognize epitopes of viral variants, thus maintaining immunity even after the emergence of viral evolution. In some cases, however, CD8+ T cells may contribute to the pathogenesis of severe COVID-19. In particular, delayed and uncontrolled, e.g., nonspecific and hyperactivated, cytotoxic CD8+ T cell responses have been linked to poor COVID-19 outcomes. In this minireview, we summarize the tremendous knowledge about CD8+ T cell responses to SARS-CoV-2 infection and COVID-19 vaccination that has been gained over the past 5 years, while also highlighting the critical knowledge gaps that remain.

## INTRODUCTION

The SARS-CoV-2 pandemic has taught important lessons about the central role of virus-specific CD8+ T cells in mediating antiviral immunity, both during natural infection and following vaccination. In contrast to antibodies, which primarily prevent viral entry, CD8+ T cells exert antiviral functions by recognizing viral peptides presented on infected cells, allowing them to directly kill the virus-infected cells and prevent further viral spread. This is particularly important for individuals with reduced or absent humoral responses. Indeed, virus-specific CD8+ T cell responses induced by infection are associated with improved survival and thus, with a protective role, in immunosuppressed individuals, who have deficient humoral and B cell immunity (1). The protective role of CD8+ T cells during viral infection has further been demonstrated in preclinical models where depletion of CD8+ T cells abrogated viral control and CD8+ T cell transfer improved viral control (2). Moreover, CD8+ T cells have been suggested to play a significant role in the control of emerging viral variants. CD8+ T cells target a broad range of epitopes from both structural and nonstructural proteins of the virus, which are often conserved across different strains (3–5). The breadth of the CD8+ T cell response together with the high level of conservation enables virus-specific CD8+ T cells to cross-recognize various viral variants (4–7), thereby mitigating the impact of mutations that may arise over time. Consequently, a robust CD8+ T cell response can be crucial in maintaining immunity and preventing severe disease, even in the face of viral evolution. Infection- and vaccine-induced CD8+ T cell responses are stable over time and are rapidly reactivated in case of reinfection, hence exhibiting robust memory responses that contribute to long-lasting immunity (8, 9). However, despite these beneficial effects, CD8+ T cells have also been implicated in the pathogenesis of severe COVID-19. They may be involved indirectly by an insufficient ability to limit viral replication and spread or directly through nonspecific effector functions that lead to the killing of uninfected cells and tissue damage. In this minireview, we highlight some of the key features of SARS-CoV-2-specific CD8+ T cell immunity during natural infection and after vaccination that contribute to both viral control and disease pathogenesis.

## ROLE OF VIRUS-SPECIFIC CD8+ T CELLS DURING SARS-COV-2 INFECTION

**Fig 1:**
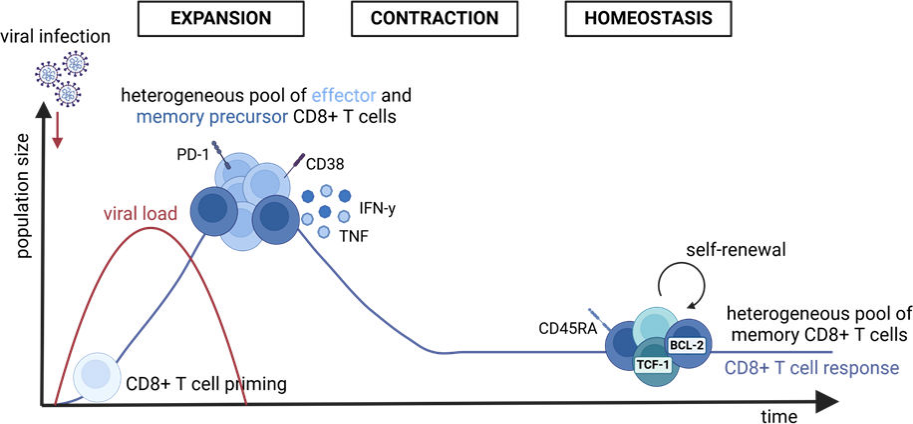
Kinetics of SARS-CoV-2-specific CD8+ T cell responses after natural infection. Early after infection, virus-specific CD8+ T cells expand, while forming a heterogeneous pool of highly activated effector CD8+ T cells and memory precursors. After viral clearance, the virus-specific CD8+ T cell response contracts, resulting in a stable pool of memory CD8+ T cells that persist long term.

**Fig 2:**
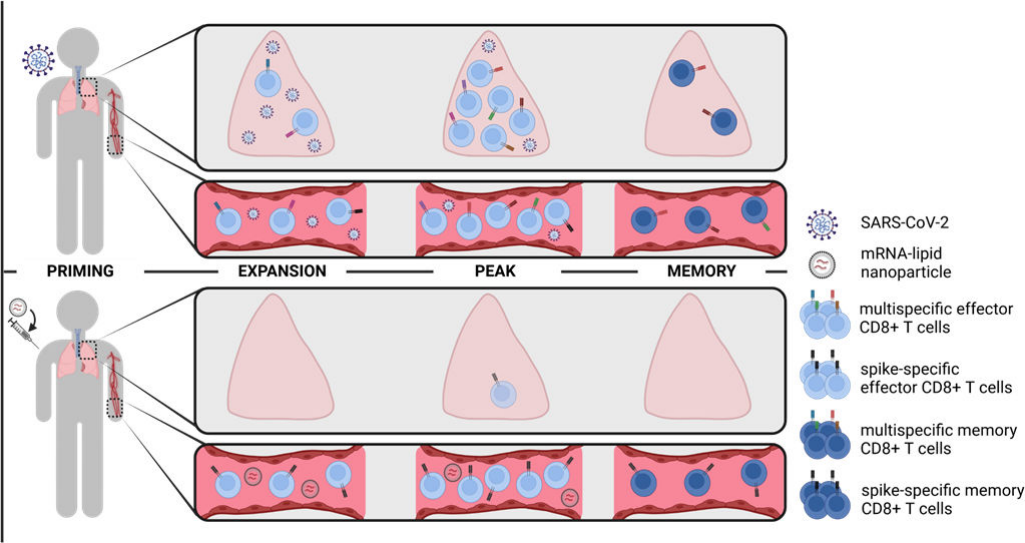
SARS-CoV-2-specific CD8+ T cell responses in lung and peripheral blood. Natural infection induces expansion of multispecific CD8+ T cells in the lung and peripheral blood, whereas (mRNA) vaccination primarily induces spike-specific CD8+ T cells in the periphery. After peak response, virus-specific memory CD8+ T cells are formed, which persist in the lungs and blood.

The COVID-19 pandemic and the tremendous efforts to understand the correlates of T-cell-mediated immune protection have yielded immense novel insights into the role of virus-specific CD8+ T cells during an acute human viral infection. In fact, by using several different methodological approaches ranging from functional and phenotypic to transcriptomic analyses, multiple groups have investigated the basic principles of SARS-CoV-2-specific CD8+ T cell immunity during acute and resolved infection. The key findings can be summarized as follows:

First, virus-specific CD8+ T cells are rapidly induced in approximately 70%–90% of COVID-19 cases within 1 month after infection and display an estimated half-life of ~200 days (10–13). By longitudinal analyses, we identified the peak of virus-specific CD8+ T cells as early as 1 week postinfection (12). At these early time points, the SARS-CoV-2-specific CD8+ T cells highly express activation markers such as CD38, CD69, or PD-1 (Fig. 1). The expression of PD-1 is thereby linked to activation and not exhaustion as these cells show no functional impairments (14). In fact, SARS-CoV-2-specific CD8+ T cells display high expression levels of effector molecules including IFN-γ, TNF, and CD107a as a surrogate for degranulation. This polyfunctional profile was present during the expansion phase (~30 days) as well as at later time points (>180 days after symptom onset) (10, 12). After the expansion phase, the virus-specific CD8+ T cell response shows a prolonged contraction phase and subsequently displays an effector memory differentiation state, as indicated by the expressions of CD45RA, CX_3_CR1, KLRG1, and CD57 (12) (Fig. 1). Although some heterogeneity has been reported in the phenotype and function of SARS-CoV-2-specific CD8+ T cells during acute viral infection, the majority of studies suggest that the SARS-CoV-2-specific CD8+ T cell immunity follows the classical T cell differentiation program described for other acute self-limiting infections. Notably, the SARS-CoV-2-specific CD8+ T cell response is multispecific and targets an estimated median of 17 targeted epitopes per individual with an immunodominance toward epitopes derived from nucleo- and membrane proteins (15) (Fig. 2).

Second, SARS-CoV-2-specific CD8+ T cells are readily detectable in the upper respiratory tract and lung tissue of infected patients, even at higher frequencies when compared to peripheral blood (16) (Fig. 2). These tissue-resident SARS-CoV-2-specific CD8+ T cells with high functionality are even present in cases where circulating virus-specific CD8+ T cells are barely detectable (17), indicating a specific enrichment at the site of the disease. Tissue-resident CD8+ T cells found in the airways of SARS-CoV-2-infected patients, who survived COVID-19, exhibited an activated and functional profile indicating their ability to kill virus-infected cells and mediate viral clearance. In line with this, a larger proportion of effector CD8+ T cells with characteristics of tissue residency were found in bronchoalveolar lavage fluid obtained from SARS-CoV-2-infected subjects with moderate compared to severe COVID-19 (18, 19). The virus-specific CD8+ T cells also appear to be long-lived at the site of infection as virus-specific memory CD8+ T cells are still detectable in the lungs, lung-associated lymph nodes, and nasal mucosa for up to 12 months after the last antigen exposure (17, 20–22) (Fig. 2). Nevertheless, it is important to note that the dynamics of emergence and persistence of virus-specific CD8+ T cells differ between anatomical sites as they are colonized by distinct T cell populations with different abilities to persist. For instance, the nasal turbinates exhibit exclusively CD8+ T cells, while the nasopharyngeal adenoids are characterized by CD4+ and CD8+ T cells (23). Moreover, the persistence of memory T cells may also differ between the upper respiratory tract and the lungs. In fact, SARS-CoV-2-specific T cells appear to reduce in the nasal fluid 3–4 months after infection (24), whereas they are detectable for more than 1 year in the nasal tissue (22) and for up to 10 months in the lungs (17).

Third, SARS-CoV-2 infection leads to the formation of memory precursor CD8+ T cells that are characterized, e.g., by the expression of classical differentiation, transcription, and pro-survival markers such as CD45RA, CD127, TCF-1, and BCL-2 (11, 12) (Fig. 1). These cells are relevant for maintaining the CD8+ T cell response and for the generation of a robust, long-lasting CD8+ T cell memory pool. Within the pool of circulating SARS-CoV-2-specific CD8+ T memory cells, different memory subsets exist, such as central memory (T_CM_), effector memory (T_EM_), and terminally differentiated effector memory expressing CD45RA (T_EMRA_) T cells (12). Among these different CD8+ T cell memory subsets, T_EMRA_ cells have been reported to be the most abundant subset (11, 25). These cells have also been associated with protection against several other viral pathogens, including influenza virus (IV), cytomegalovirus (CMV), Epstein–Barr virus (EBV), and human immunodeficiency virus (HIV) (26–29). The virus-specific CD8+ T cell memory remains stable over time and is still present long after infection (30) (Fig. 1). These cells are characterized by a transcriptional signature that marks long-lived, circulating human memory CD8+ T cells following an acute viral infection (31). SARS-CoV-2-specific memory CD8+ T cells are fully functional and similar to other viral infections, such as influenza virus (12), even though they are present at a slightly lower frequency.

Lastly, and most importantly, early SARS-CoV-2-specific CD8+ T cell responses are associated with a reduced disease severity and an asymptomatic course of primary infection (32–34). In fact, the emergence of a rapid, robust, and polyfunctional CD8+ T cell response in the periphery has been shown to correlate with lower peak viral loads, rapid viral clearance, and a better overall clinical outcome (35). One study even observed a higher cytokine production per T cell in asymptomatic infection, affirming their high functionality and antiviral efficacy (36). The critical role of virus-specific CD8+ T cells in mediating antiviral immunity has also been demonstrated by their presence in individuals exposed to SARS-CoV-2 who did not become infected, suggesting that early T cell responses can facilitate rapid viral clearance before seroconversion (34, 37, 38). Conversely, more severe cases of COVID-19 have been associated with a delayed induction and a lower frequency of SARS-CoV-2-specific CD8+ T cells in the peripheral blood (39, 40) and lung tissue (19, 41), as well as a disproportional secretion of inflammatory cytokines (36). Hence, the emergence of an early and functionally competent virus-specific CD8+ T cell response seems critical for a mild course of disease. This conclusion is further supported by the finding that protective CD8+ T cell immunity is present in immunosuppressed patients with reduced humoral responses and B cell deficiency. CD8+ T cell responses are also associated with an improved survival of SARS-CoV-2-infected patients with hematologic cancers who have impaired humoral responses and are treated with anti-CD20 therapy (1). Noteworthily, unlike antibody-mediated protection, CD8+ T cells maintain their protective effects regardless of which epitope of the viral proteins they target or where these epitopes are located within the virus. Furthermore, the protective efficacy of CD8+ T cells in SARS-CoV-2 infection has been shown in macaques, where depletion of CD8+ T cells after SARS-CoV-2 infection caused increased viral loads following re-challenge with SARS-CoV-2 (42).

## CD8+ T CELL RESPONSES AFTER VACCINATION

The development of efficient COVID-19 vaccines and subsequent international vaccination campaigns have successfully reduced the risk of primary SARS-CoV-2 infection and severe disease. Many different vaccine platforms have been tested in humans, and several of them have been approved. The most widely used vaccines are from Pfizer-BioNTech (Comirnaty), Oxford-AstraZeneca (Vaxzevria), Moderna (Spikevax), Johnson & Johnson (Jcovden), Sinopharm BIBP (Covilo), Sinovac (CoronaVac), and Novavax (Nuvaxovid) and represent mRNA-, adenoviral vector-, inactivated virus-, and protein-based vaccine technologies (https://covid19.trackvaccines.org/agency/who/). The different vaccine platforms elicit distinct T cell responses. For example, the inactivated virus vaccine induces robust CD4+ T cell responses in most individuals, while CD8+ T cell responses are hardly detectable (43). Still, a key advantage of this vaccine is its ability to induce T cell responses not only against the spike protein but also against all other viral proteins, many of which tend to be more conserved across different viral variants (44, 45). In contrast, mRNA and adenoviral vector vaccines that are used in most countries (https://covid19.trackvaccines.org/agency/who/) are solely based on the spike protein and, therefore, elicit only spike-specific T cell responses (Fig. 2). These responses, however, are robust and mediated by both CD4+ and CD8+ T cells, in most individuals (46, 47).

Early studies on SARS-CoV-2-specific CD8+ T cells following vaccination with mRNA- and adenoviral vector-based regimens have demonstrated a rapid induction of spike-specific CD8+ T cells in most individuals as early as 5–7 days after the initial (prime) immunization (Fig. 2). Indeed, by performing a longitudinal analysis starting at the baseline of prime vaccination until several months after boost, we observed in agreement with other reports (48) a rapid and stable mobilization of spike-specific CD8+ T cells by SARS-CoV-2 mRNA vaccination at a time point when neutralizing antibodies were hardly detectable (9). The peak mobilization of neutralizing antibodies is first detectable after boost, most likely reflecting the maturation of this response in the secondary lymphoid organs before they are released to the circulation. A strong induction of spike-specific CD8+ T cells following mRNA vaccination has also been observed in immunosuppressed patients, such as those receiving anti-CD20 therapy and liver transplant recipients, despite their impaired humoral responses (49–52). Although the frequencies of the vaccine-induced CD8+ T cell responses may be lower in immunosuppressed compared to immunocompetent individuals, they are still functionally competent in most individuals (51, 53). Indeed, the functional capacity of spike-specific CD8+ T cells after mRNA vaccination is comparable to those induced by natural infection, albeit slight differences in the phenotype and kinetics. For example, a lower expression of the activation marker CD38 has been observed on early memory spike-specific CD8+ T cells after vaccination compared to natural infection (9, 12), indicating enhanced activation after infection with different kinetics. In line with this, a steeper contraction of the spike-specific CD8+ T cell response has been reported after vaccination compared to the prolonged contraction phase detected after natural infection (9, 12). Most likely, the differential route and duration of antigen contact contribute to these differences.

Importantly, as in natural infection, vaccination alone is also capable of inducing tissue-resident SARS-CoV-2-specific CD8+ T cells in nasal mucosal tissues, where they can rapidly respond to subsequent infection (22). In line with this, the first appearance of vaccine-induced SARS-CoV-2-specific CD8+ T cells coincides with the first protective clinical effect that can be observed within 12 days after the first vaccination, clearly supporting a potential protective role of virus-specific CD8+ T cell immunity (54). This is also supported by studies performed in nonhuman primates. Indeed, vaccinated macaques with strong virus-specific CD8+ T cell responses are able to control infection in the absence of SARS-CoV-2-specific neutralizing antibodies (55). The important role of vaccine-induced virus-specific CD8+ T cells in viral clearance is further supported by a study in vaccinated macaques where depletion of CD8+ T cells prior to infectious challenge prolonged viral infection (2). Moreover, vaccine failures in experimental Omicron challenge studies were linked to a lack of CD8+ T cell immunity, despite the presence of moderate antibody titers (56), further emphasizing the critical protective role of CD8+ T cells in viral infections.

The virus-specific CD8+ T cell response can be further enhanced by a booster vaccine. However, variations in the frequency of virus-specific CD8+ T cells have been observed depending on whether a homologous or heterologous booster vaccine was administered. Individuals who received an mRNA vaccine as a prime vaccination and a boost with the adenoviral vector-based vaccine Jcovden (heterologous booster) elicited higher spike-specific CD8+ T cell responses compared to those who received homologous booster (57). Similarly, individuals who received a prime vaccination with an adenoviral vector-based vaccine (Vaxzevria) and were boosted with an mRNA vaccine exhibited stronger spike-specific CD8+ T cell responses compared to individuals who received a homologous prime/boost vaccine regimen (58). Still, the overall effectiveness in protecting from severe disease is comparable between heterologous versus homologous regimens (59). In-depth studies of mRNA-vaccinated healthy individuals demonstrated that boosting with a second, third, or fourth dose of the mRNA vaccine results each time in a robust expansion of highly activated spike-specific effector CD8+ T cells that peak around 5–6 days after the respective boosting. This rapid expansion in spike-specific CD8+ T cells lasts approximately 30–60 days and is followed by a subsequent contraction phase, in which CD8+ T cell frequencies decrease until they reach frequencies similar to those observed before the boost vaccination (8, 9). Importantly, spike-specific CD8+ T cells retain their functional capacity throughout booster vaccination. This has been shown for both healthy and immunosuppressed individuals (8, 9, 51). Overall, these findings underline that vaccination induces fully functional CD8+ T cells with robust recall responses.

Vaccination leads to the stable formation of CD8+ T cell memory, as has been reported for natural infection (Fig. 2). However, the distribution of the different memory subsets varies slightly. For example, while higher numbers of early differentiated memory subsets, such as early differentiated, central memory, and transitional memory T cells, emerge after natural infection, higher frequencies of effector memory CD8+ T cells are present after vaccination (9). Furthermore, mRNA vaccination and natural infection induce the formation of stem cell-like memory CD8+ T cells (T_SCM_). These cells have previously been identified in individuals who received the yellow fever virus vaccine, which is highly effective and induces long-term immunity in humans (60). T_SCM_ have self-renewal capacities and are, therefore, essential for the generation of long-lasting CD8+ T cell immunity. Spike-specific CD8+ T_SCM_ cells are already detectable after prime vaccination (8, 61) and remain stable in frequency and phenotype over time and throughout further booster vaccinations (8). This indicates that vaccination induces the formation of a long-lived CD8+ T cell memory pool. Stable CD8+ T cell memory formation has been observed for at least 6–8 months after both vaccination regimens, adenoviral vector-based vaccination (62) and mRNA vaccination (63, 64). Yet, differences in the frequencies of memory CD8+ T cells may occur in individual cases. For example, reduced long-term cellular responses have been observed in high-risk populations such as elderly people and immunosuppressed individuals (51, 65, 66). However, although the spike-specific CD8+ T cell frequencies are reduced, they appear to remain stable over time (51), suggesting long-term persistence even in high-risk populations.

## BREAKTHROUGH INFECTION AND HYBRID IMMUNITY

Virus-specific CD8+ T cells also play an important role in the context of breakthrough infection that is defined as an infection occurring in individuals despite prior vaccination against the same pathogen. Although vaccine-induced virus-specific CD8+ T cells do not necessarily protect vaccinated individuals from infection (67, 68), they still may play a central role in limiting disease severity (67–69) by rapidly responding to breakthrough infection (8) and thereby contributing to viral containment and limiting disease severity and duration. During the course of a breakthrough infection, memory T cells, formed by prior vaccination, are reactivated, rapidly expand, and respond in greater magnitude to the antigenic trigger compared to naïve T cells (70). These memory T cells usually target epitopes, which are shared with the previously encountered pathogen, while additional responses against new epitopes may also be initiated (71). In other viral infections, such as new influenza or dengue virus variants, only modest immune responses are generated against new epitopes, which may be associated with a negative clinical outcome (72). This immunological phenomenon is called “original antigenic sin” (OAS) and implies that when the epitope varies slightly, the immune system may rely on “suboptimal” memory. In the context of SARS-CoV-2, however, several studies have demonstrated that breakthrough infection leads to the induction of robust T cell responses including both pre-existing and *de novo* T cell responses (24, 73, 74). These responses are often directed against multiple epitopes derived from proteins that are not included in the vaccines. In addition, breakthrough infection with a viral variant elicits *de novo* CD8+ T cell responses not only against the infecting variant but also against newly emerging SARS-CoV-2 subvariants (74). Thus, in the case of COVID-19 vaccination, OAS does not seem to affect the robustness of the T cell response. Instead, the immunity induced by the combination of both vaccination and infection, defined as hybrid immunity, is beneficial in the case of subsequent infection. In fact, as also discussed above, the multispecific T cell response induced by hybrid immunity is especially important against emerging viral variants as they offer protection against a wider range of potential epitopes (3, 24, 73). Hybrid immunity also leads to robust and durable T cell responses in the lung mucosa (75), which have been linked to protection at the site of infection (24). Consequently, hybrid immunity induces improved protection against subsequent infection, when compared to either only infection- or vaccination-induced immunity (76, 77).

## CROSS-RECOGNITION AGAINST OTHER CORONAVIRUSES OR VARIANTS OF CONCERN

Early in the pandemic, several studies reported the presence of cross-reactive T cells in individuals who had not been exposed to SARS-CoV-2. These cross-reactive memory T cells recognize epitopes of other coronaviruses, particularly of common cold coronaviruses and SARS-CoV-1 that share sequence homology with SARS-CoV-2 (12, 13, 78–80). Cross-reactivity has been described for both CD4+ and CD8+ T cells; however, it seems to be more prevalent among the CD4+ T cell compartment (81). The biological relevance of cross-reactive T cells has been shown in different scenarios. For example, their presence has been linked to a milder course of disease (33, 82) and even the prevention of SARS-CoV-2 infection in naïve individuals (83, 84). Indeed, cross-reactive T cells have been described in SARS-CoV-2 exposed healthcare workers and family members, who remained PCR- and seronegative despite viral exposure (34, 85). The potential role of cross-reactive memory CD8+ T cells as a first line of protection from infection is further supported by their presence in oropharyngeal lymphoid tissues of unexposed individuals (86). Not surprisingly, pre-existing cross-reactive T cells have also been suggested to be important after vaccination as stronger and more durable T cell responses following COVID-19 vaccination have been observed in individuals with pre-existing cross-reactive T cells (64, 87).

**Fig 3:**
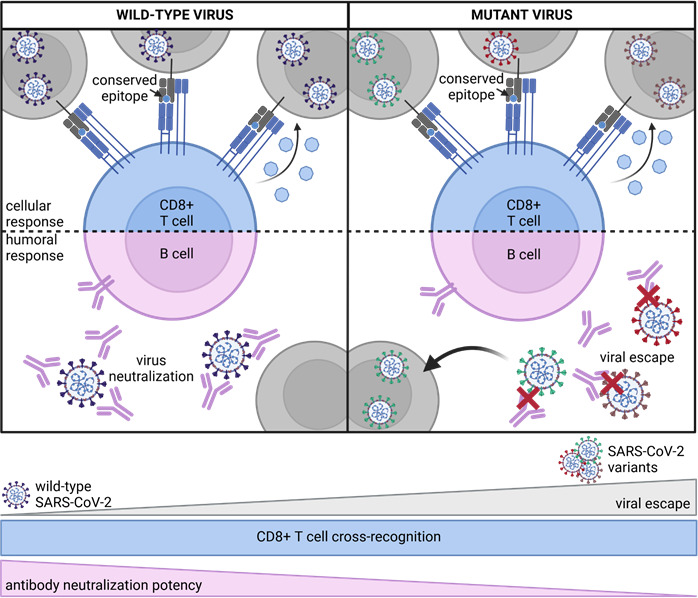
SARS-CoV-2-specific CD8+ T cell cross-recognition. SARS-CoV-2-specific CD8+ T cells target mainly conserved epitopes, enabling them to cross-recognize viral variants. In contrast, antibodies target surface proteins that have been more susceptible to mutation in the viral variants, leading to viral escape and reduced neutralization potency.

The most important role of cross-reactive SARS-CoV-2-specific CD8+ T cells is most likely in combating against viral variants of concerns (VOC) that emerged shortly after the initial vaccination rollout. These variants, including Alpha, Beta, Gamma, and later Omicron, exhibit several mutations that affect viral transmission, disease severity, and immune escape. The virus with the highest number of mutations, Omicron, became quickly dominant starting late 2021/early 2022 and showed enhanced evasion of humoral responses that were induced by previous infection or vaccination (88). In contrast to B cell epitopes, virus-specific T cell epitopes appear to be less affected by these mutations and are largely conserved among the different variants (4, 5) (Fig. 3). Thus, epitopes derived from VOCs can still be recognized by virus-specific CD8+ T cell responses primed by the ancestral strain (4–7, 89). In fact, although Omicron has more than 30 mutations in the spike protein (90), vaccine-induced spike-specific CD8+ T cells show high cross-reactivity (over 80%) against this variant (91, 92). This high level of cross-reactivity can be explained by several factors that make T cell epitopes less prone to mutations. One key factor is HLA polymorphism. The vast diversity of HLA genes in the human population enables the presentation and recognition of thousands of different epitopes. As a result, mutations of individual T cell epitopes would only confer an advantage to the virus in a small fraction of the population. Moreover, this diversity reduces the selective pressure on the virus to mutate these T cell epitopes. Another important factor is that the T cell epitopes are often located in regions of viral proteins that are crucial for viral function and replication (3). Hence, mutations in these regions would compromise viral fitness, making them less likely to persist (93).

## PATHOGENIC ROLE OF CD8+ T CELL IMMUNITY

**Fig 4:**
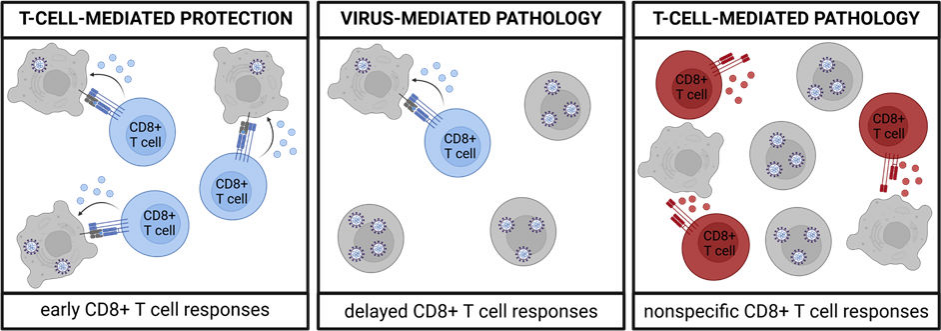
Protective and pathological role of CD8+ T cells in acute viral infection. Early and high frequencies of SARS-CoV-2-specific CD8+ T cell responses mediate rapid viral clearance, resulting in protection from severe disease (left). Delayed and reduced frequencies of SARS-CoV-2-specific CD8+ T cell responses fail to appropriately control viral replication and spread, leading to virus-induced pathology (middle). Nonspecific and uncontrolled effector functions lead to the killing of uninfected cells, but not to viral control, resulting in tissue damage (right).

Next to their antiviral effects, virus-specific CD8+ T cells can also contribute to disease severity, e.g., a more severe clinical course of COVID-19 (94–96). The exact mechanisms contributing to a more pronounced pathogenic effect of SARS-CoV-2-specific CD8+ T cells are not well understood. It is possible that they may indirectly contribute to the pathogenesis of the disease by, e.g., delayed induction and reduced frequencies (19, 39–41, 97), most likely leading to failure to appropriately limit viral replication (Fig. 4). In addition, impaired viral control in the early stages of infection causes increased viral replication that results in an enhanced and dysregulated immune response later in the infection. In line with this, delayed, but prolonged and excessive activation of CD8+ T cells has been observed in patients with severe COVID-19 (94, 95, 97). Furthermore, CD8+ T cells can also directly contribute to pathology by exerting their effector functions in an uncontrolled and nonspecific manner (immunopathology; Fig. 4). During viral infection, CD8+ T cells rapidly produce proinflammatory cytokines upon antigen encounter to mediate efficient viral clearance. However, if these effector functions are unrestricted, they can cause tissue damage and inflammation. Indeed, a higher frequency of bronchoalveolar lavage cytotoxic CD8+ T cells has been linked to epithelial damage and airway disease in individuals with persistent symptoms after acute COVID-19 (98). Moreover, tissue damage can also be caused by misdirected cytotoxicity of non-SARS-CoV-2-specific CD8+ T cell activation that provokes apoptosis of uninfected cells and, thus, may also contribute to the pathogenesis of severe disease (Fig. 4). This TCR-independent T cell activation can be induced, e.g., by the complement system (99) or by a proinflammatory environment (100). In the latter case, the non-SARS-CoV-2-specific T cells are readily activated in a TCR-independent, bystander manner by proinflammatory cytokines, such as type I IFNs (100). Due to their rapid induction, bystander-activated T cells can be present even before the induction and development of antigen-specific T cell responses, which usually takes a few days. In this way, bystander-activated T cells may play an important role in the early viral defense. Yet, in cases of excessive and uncontrolled stimulation, they may also contribute to immunopathology. These bystander-activated T cells can be identified by the expression of activation markers (e.g., CD38 and HLA-DR), cytotoxic molecules (e.g., granzyme B), and the natural killer cell-activating receptor NKG2D (101). These cells have been found in patients with severe COVID-19 (97), supporting their possible role in mediating disease severity. Furthermore, CD8+ T cells have also been suggested to be involved in the pathogenesis of long COVID. However, findings on this topic are conflicting. Some studies have reported the presence of exhausted SARS-CoV-2-specific CD8+ T cells in individuals who suffer from long COVID (102), while others have identified hyperactivated CD8+ T cells with augmented granzyme B and IFN-y production (103, 104). In contrast to these findings, another in-depth study observed similar SARS-CoV-2-specific T cell responses, memory formation, and TCRαβ clonotypes in people with long COVID compared to non-long COVID individuals (105), indicating that SARS-CoV-2-specific T cells may not play a significant role in the pathogenesis of long COVID. Alternatively, it has been hypothesized that immune responses to recently reactivated, latently persisting viruses, such as EBV, may be involved in the development of long COVID symptoms (106). A further possible hypothesis is that immunosuppressive KIR+ CD8+ T cells may contribute to disease pathogenesis by hampering virus-specific immune responses. Indeed, higher frequencies of KIR+ CD8+ T cells have been detected in patients with severe COVID-19 (107). Nevertheless, despite extensive research, the exact role of CD8+ T cells in long COVID remains to be determined.

## CONCLUSIONS AND OPEN QUESTIONS

SARS-CoV-2 has been an excellent model for studying antiviral CD8+ T cell immunity in humans, confirming fundamental principles of CD8+ T cell responses in humans, while also revealing novel aspects of their role in acute viral infection. Even though much has been learned, several key questions still remain unanswered. For instance, memory CD8+ T cells with stem cell-like characteristics have been detected in the peripheral blood after natural infection and vaccination. So far, these memory CD8+ T cells have only been studied for 12 months after infection and 6–8 months after vaccination. Nevertheless, it can be assumed that SARS-CoV-2 induces a similarly long-lasting T cell immunity, as described after SARS-CoV-1 infection, where virus-specific T cells were still detectable 17 years after infection (80). However, as SARS-CoV-2 persists as an endemic pathogen, the question remains open as to how this CD8+ T cell immunity is shaped and maintained in terms of repertoire and function by frequent exposure to the virus. Furthermore, the factors that influence the kinetics and magnitude of SARS-CoV-2-specific T cell responses and their dysregulation during severe disease still need to be determined. The complex interaction of CD8+ T cells with other components of the immune system, e.g., with the innate immune system, is also only little understood. Finally, the role of CD8+ T cells in the pathogenesis of long COVID remains a crucial area of investigation, and future studies need to reveal whether long COVID is caused by prolonged activation of immune cells due to dysregulation, antigen persistence, or by autoimmune phenomena. In conclusion, SARS-CoV-2 continues to serve as a valuable model for studying CD8+ T cell immunity in humans and remains especially pertinent for the development of vaccine strategies for other emerging viruses.
